# Global Coordination and Standardisation in Marine Biodiversity through the World Register of Marine Species (WoRMS) and Related Databases

**DOI:** 10.1371/journal.pone.0051629

**Published:** 2013-01-09

**Authors:** Mark J. Costello, Philippe Bouchet, Geoff Boxshall, Kristian Fauchald, Dennis Gordon, Bert W. Hoeksema, Gary C. B. Poore, Rob W. M. van Soest, Sabine Stöhr, T. Chad Walter, Bart Vanhoorne, Wim Decock, Ward Appeltans

**Affiliations:** 1 Leigh Marine Laboratory, University of Auckland, Auckland, New Zealand; 2 Muséum National d'Histoire Naturelle, Paris, France; 3 The Natural History Museum, London, United Kingdom; 4 Smithsonian Institution, Washington, District of Columbia, United States of America; 5 Aquatic Biodiversity & Biosecurity, National Institute of Water & Atmospheric Research, Wellington, New Zealand; 6 Naturalis Biodiversity Center, Leiden, The Netherlands; 7 Museum Victoria, Melbourne, Victoria, Australia; 8 Swedish Museum of Natural History; Department of Invertebrate Zoology, Stockholm, Sweden; 9 Flanders Marine Institute, Ostend, Belgium; 10 Intergovernmental Oceanographic Commission of UNESCO; IOC Project Office for IODE, Ostend, Belgium; Institute of Marine Research, Norway

## Abstract

The World Register of Marine Species is an over 90% complete open-access inventory of all marine species names. Here we illustrate the scale of the problems with species names, synonyms, and their classification, and describe how WoRMS publishes online quality assured information on marine species.

Within WoRMS, over 100 global, 12 regional and 4 thematic species databases are integrated with a common taxonomy. Over 240 editors from 133 institutions and 31 countries manage the content. To avoid duplication of effort, content is exchanged with 10 external databases. At present WoRMS contains 460,000 taxonomic names (from Kingdom to subspecies), 368,000 species level combinations of which 215,000 are currently accepted marine species names, and 26,000 related but non-marine species. Associated information includes 150,000 literature sources, 20,000 images, and locations of 44,000 specimens. Usage has grown linearly since its launch in 2007, with about 600,000 unique visitors to the website in 2011, and at least 90 organisations from 12 countries using WoRMS for their data management.

By providing easy access to expert-validated content, WoRMS improves quality control in the use of species names, with consequent benefits to taxonomy, ecology, conservation and marine biodiversity research and management. The service manages information on species names that would otherwise be overly costly for individuals, and thus minimises errors in the application of nomenclature standards. WoRMS' content is expanding to include host-parasite relationships, additional literature sources, locations of specimens, images, distribution range, ecological, and biological data. Species are being categorised as introduced (alien, invasive), of conservation importance, and on other attributes. These developments have a multiplier effect on its potential as a resource for biodiversity research and management. As a consequence of WoRMS, we are witnessing improved communication within the scientific community, and anticipate increased taxonomic efficiency and quality control in marine biodiversity research and management.

## Introduction

### The taxonomic challenge

Taxonomy, the science of discovering and naming species, must have been one of the earliest human activities. Names are given to species when they are recognised as distinctive and important to human culture, whether because of their value for food, ecology (e.g. habitat forming), recreation, potential hazards they may pose, and as objects of admiration. Today, biological diversity is threatened with mass extinction due to climate change, over-hunting, species introductions (especially to islands), and habitat loss [1], [2], [3]. Indeed, some authors worry that the rate of species extinction is exceeding their rate of scientific description [4]. Species are the fundamental practical units of biology, and thus the accurate naming of species is critical for all biology [5]. However, progress in their description and classification is slower than for elements in chemistry and particles in physics simply because there are thousands times more species than of these non-biological units. Thus a major challenge in taxonomy is to accelerate the process of species discovery.

Local and regional species checklists are in demand for conservation and fisheries management, ecological surveys, and training in marine ecology and environmental management. However, these lists are inevitably compromised by either by not being updated by experts, by inheriting past misuse of names, by using the same name for dissimilar species in different locations, by using differing names for the same species in different regions, or, finally by combinations of these problems. The simplest solution to this confusion would be a single authoritative world register routinely updated by experts that is freely accessible on the World Wide Web. The lack of such a world register partly reflected the local and regional focus of biology in the past. It also reflected the high diversity of species, and the hundreds of publications in which they are described, all problems that made collating a checklist beyond the capability of even a modest group of scientists.

Without standardised names for species, the management and use of biodiversity is compromised [6]. Even within different languages and countries, species may have different common or vernacular names, and the same names applied to different species. For example, the ‘common blue’ is a damselfly, a butterfly or a thistle in the UK. The ‘green sea urchin’ is *Psammechinus miliaris* (Müller, 1771) in the North-East Atlantic, but in the North-West Atlantic it is the commercial species *Strongylocentrotus droebachiensis* (Müller, 1776); note that the latter was not only described from Europe, but remains common there.

Linnaeus's binominal system for naming species in Latin, taken to have commenced in 1753 for Botany and 1758 for Zoology [7], overcame the problem of vernacular names in different languages but introduced its own set of problems. In the Annual checklist of the Catalogue of Life (CoL) [8] the name *vulgaris* occurs 1,106 times and is used for many plants (including seaweeds, conifers, legumes), insects (including flies, aphids, weevils, fleas, grasshoppers, lepidopterans, wasps), octopus, starfish, crustaceans, bacteria, viruses, fish, and reptiles. Even when synonyms are excluded, it occurs 382 times as ‘Accepted Names’. Similarly, *virginea* is the specific epithet of a rush (plant), mollusc, sea squirt, fly, weevil, butterfly, and several fungi, and occurs 92 times and 52 times under Accepted Names. The same words have been used for different genera, e.g. *Morus* is a genus of marine bird (the gannet) and the mulberry plant; *Crepis* a genus of Bryozoa and a composite plant; *Sphenopus* is a zoanthid (Cnidaria: Anthozoa: Hexacorallia: Zoantharia: Sphenopidae) and a plant; and *Ficus* is a genus of gastropod and fig tree. Other names are used as both genus names and specific epithets, e.g. the name *Veronica* is a genus of plants (speedwells), and the specific epithet of a species of butterfly and a legume. Generic names from different kingdoms can also be similar: *Cantharellus* is a genus of mushrooms (terrestrial fungi belonging to the Basidiomycota: Agaricomycetes: Cantharellales: Cantharelaceae) but also of mushroom corals (Cnidaria: Anthozoa: Hexacorallia: Scleractinia: Fungiidae); *Turbinaria* is a scleractinian genus (Dendrophylliidae) occurring on Indo-Pacific coral reefs with a genus of brown alga of the same name (Ochrophyta: Phaeophyceae: Fucales: Sargassaceae). Species can also be named for people and geographic places, further complicating searches for information unless they are clearly context specific. In these cases, confusion is usually avoided because the genus name must always be used in combination with the ‘specific epithet’ and it is unusual for the same genus and specific epithet to be combined (but see below under Homonyms). However, unintentionally, species have often been given more than one scientific name, or the same name may have been used for more than one species, a species may have been described in one genus and later moved to one or more other genera, or often names are misspelled.

Choosing the correct name is governed by international codes, the International Code of Zoological Nomenclature [9], [10], the International Code of Nomenclature for algae, fungi, and plants [11], [12] and the International Code of Nomenclature of Bacteria [13]. Future discoveries often find that what was once considered one species is now several, so the application of a name may change over time, and it may be re-classified. New genus assignations can confuse users because it appears to be a new name often for a well-known species. The rules of nomenclature also require the specific epithet to agree in gender with the genus, so a change in genus may mean that the ending of the specific epithet changes (e.g. –*um* to –*a*). Additionally, the higher classifications of life have significantly changed in recent decades owing to discoveries of relationships. New kingdoms and phyla have been recognized and more than one phylum has been merged to another (e.g., the formerly recognized phyla Vestimentifera, Pogonophora, Echiura and Sipuncula are now included in the phylum Annelida), and groups of species re-allocated within classes, orders and families. For example, the Microsporidia were transferred from the protists (protozoans), or animals, to the fungi [14], [15], [16]. Changing species names, especially reclassification, is not a fault of the system but reflects the nature of discovery. Indeed, we may know most species in Europe [17], [18] (but see [19]), and amongst vertebrates and higher plants, but one third to four fifths of all species may remain to be described [20], [21], [22], [23]. Thus we expect more species to be discovered, species reclassified into different genera and families, and some currently recognized species to be synonymised.

To further standardise species nomenclature, all new bacteria species must be described in a particular journal [13], [24], and from 2013 scientific names of fungi will have to be registered in a recognized repository (e.g., MycoBank) [25]. In contrast, animal and plant species can be named in any print publication and no mandatory register of names exists. Having an online inventory of all accepted species names is an essential precursor to such a registration system for animal and plant names. The International Plant Names Index provides such a register for flowering plants [26] of which few occur in the ocean. The International Commission on Zoological Nomenclature, which is responsible for the International Code of Zoological Nomenclature, has established ‘ZooBank’, as an online registration system for animal names [27], [28], [29]. A further opportunity is for zoologists to standardise the nomenclature of particular taxa by restricting availability of names to a ‘List of Available Names’, as proposed for the 3,570 names in the Phylum Rotifera [30]. This could help taxonomy by making names applied to uncertain species (e.g., species poorly described and/or without type specimens) unavailable and thus no longer usable. Already having an expert validated list of species names is a prerequisite for such an initiative.

### Synonyms

Synonyms arise where different specimens that later are found to be the same species have been given different names, i.e. subjective (in zoology) or heterotypic (in botany) synonyms. The fraction of junior synonyms has been reported to be: 7 to 80% (32% overall) in different insect orders and families [31], [32]; 37% for molluscs [33]; 81% in European freshwater fish [34]; 27% for fossil North American mammals [35]; 33% to 88% for groups of seed plants [36], [37]; and 50% for marine fish [38]. At first, it seems that the most popular taxa, which are most intensively studied and by most people, have more synonyms. However, it is possible that similar proportions of synonyms occur in other taxa that are less well studied. Furthermore, some of these taxa may be very species-rich. The only way to discover these problems is for specialists to revise the taxonomy of each group of species, including re-examining type specimens, usually more thoroughly describing species (including genetic analysis) to avoid future confusion. A first step in a taxonomic revision is to review a list of species named and ask whether some may be synonyms.

Synonyms can also be discovered for taxa above the species level, resulting in changed classifications of species. For example, Johnson et al. [39] found that three families of fish, two known only from the deep-sea (>1,000 m), namely (1) bignose fish (Megalomycteridae Myers & Freihofer, 1966), (2) whalefish (Cetomimidae Goode & Bean, 1895), and (3) the shallow-water (<200 m) hairy and tape-fish (Mirapinnidae Bertelsen & Marshall, 1956), represented males, females and juveniles of just one family. Thus two families were subsumed as synonyms of the first described family. However, synonyms are more common at the species level. Male and female cuckoo wrasse look very different and Linnaeus described them under two different names in the same book, namely *Labrus mixtus* Linnaeus, 1758 and *L. bimaculatus* Linnaeus, 1758, and until recently both names were in common use. The distinctive and widely known sperm whale *Physeter macrocephalus* Linnaeus, 1758 has been described as 19 different species: three each from Linnaeus in 1758, Bonaterre in 1789, Lacépède in 1804, and Gray in 1846, 1850 and 1856; two from Borowski in 1780; and one each from G. Cuvier, Kerr, Desmoulins, Fleming and Risso [40]. However, even when scientists have clarified synonyms, old names still exist in the past literature so a reader needs to know which names may have been used for a species. One of the most popular fish in research and aquaculture, the rainbow trout, was known as *Salmo gairdneri* Richardson, 1836, but is correctly named *Oncorhynchus mykiss* (Walbaum, 1792), an older available name. A search of Google Scholar in 2009 found 39,000 citations of the incorrect name and 18,000 of the correct one; in 2012, 38,900 and 60,600 hits respectively (276,000 and 1,050,000 in Google). A sponge widely used in medical research into cell biology and cancer is widely named *Microciona prolifera* (Ellis & Solander, 1786) but should be called *Clathria prolifera* (Ellis & Solander, 1786). In this case, the species epithet is unchanged but the genus to which it belongs has been changed. Thus, information about this species needs to be sought under both names.

### Homonyms

Homonyms are identical accepted names applied to unrelated species. Examples are *Paridotea munda* Hale, 1924 and *Paridotea munda* Nunomura, 1988, both similar isopods, one in Australia and the second in Japan. As yet, a replacement name has not been proposed for the second usage. Homonyms exist within marine species, and between marine and non-marine species (Table 1). In many cases the names can be distinguished if the authority and year of description are included in the citation. Thus most journals require that the species name includes the authority and year of publication.

**Table 1:** Examples of the same names being used for different species (including a marine species) found by Rees [114].

Species	Kind of marine species	Non-marine species
*Alcyonium bursa*	cnidarian, alga and sponge	–
*Asterina gibbosa*	cushion star	fungus
*Coryne dubia*	cnidarians (hydroid)	fungus
*Culcita novae-guineae*	starfish	fern
*Cynthia carnea*	sea squirt	butterfly
*Dilophus crenulatus*	brown alga	fly
*Dilophus crinitus*	brown alga	fly
*Elachista pusilla*	brown alga	moth
*Eulalia aurea*	polychaete worm	grass
*Ficus elegans*	snail	fig tree
*Phaseolus ovatus*	bivalve	plant (pea)
*Polysiphonia tuberosa*	anemone, red alga	–
*Sargus fasciatus*	fish	fly
*Sphaerococcus durus*	red alga	hemipteran bug
*Torresia australis*	fish	reptile (gecko)
*Trentepohlia mirabilis*	green alga	crane fly
*Trentepohlia setifera*	green alga	crane fly
*Verrucaria rubra*	red alga	fungus
*Zygaena erythraea*	fish	moth
*Zygaena vulgaris*	fish	moth

These homonyms can be distinguished if the author and year of description are included after the name, because it is highly unlikely for an author to describe two different species with the same name in the same year.

The same name may be used for different genera. Marine examples include *Duplicaria* Dall, 1908 [Gastropoda] and *Duplicaria* Vine, 1972 [Polychaeta]; *Luetkenia* Duncan, 1878 [Ophiuroidea] and *Luetkenia* Claus, 1864 [Copepoda]; and *Acanthopharynx* Marion, 1870 [Nematoda] and *Acanthopharynx* Reisinger, 1924 [Platyhelminthes]. Replacement names must be proposed for the more junior name if they occur with the same code of nomenclature. One example is the case of *Singula* Blazewicz-Paszkowycz, 2005, a new name for the tanaidacean *Singularia* Blazewicz-Paszkowycz, 2005 and *Biuncus* Huys, 1995 a replacement name for *Singularia* Huys, 1995, both preoccupied by *Singularia* Arenberger, 1988, a moth.

Some accepted species names may be so similar to each other that they resemble misspelled homonyms and may cause confusion as well, such as the solitary ascidians *Polycarpa aurata* (Quoy & Gaimard, 1834) and *P. aurita* (Sluiter, 1890), or the shrimp genera *Allopontonia* Bruce, 1972, and *Altopontonia* Bruce, 1990. If they are all included in a common database then these distinctions become more apparent and reduce confusion. Thus to find information on a species one needs to know which names may be in fact referring to the same species. When a comprehensive review of a species is undertaken, a search on synonyms, misspellings and homonyms is required.

### Misspellings

Misspellings abound in the literature and are perpetuated because authors neither check the original descriptions nor even validated lists of names when available. Some misspellings are not surprising considering the similarities and peculiarities of some accepted species names. For example, *Acipenser oxyrinchus* Mitchill, 1815, *Amblycirrhitus oxyrhynchos* (Bleeker, 1858), *Cheilinus oxyrhynchus* Bleeker, 1862, *Arnoglossus oxyrhynchus* Amaoka, 1969, *Coregonus oxyrinchus* (Linnaeus, 1758), *Cestraeus oxyrhyncus* Valenciennes, 1836, *Dipturus oxyrinchus* (Linnaeus, 1758), *Facciolella oxyrhyncha* (Bellotti, 1883) and *Himantura oxyrhyncha* (Sauvage, 1878), *Rhynchopelates oxyrhynchus* (Temminck & Schlegel, 1842) are nine species of fish; and the amphipod *Westwoodilla oxyrhyncha* Bulycheva, 1952 stomatopod *Raoulserenea oxyrhyncha* (Borradaile, 1898) and decapod *Oxyrhynchaxius* Parisi, 1917 comprise two species and one genus of crustaceans with similar names. With such similar-sounding and -spelt specific epithets it is no wonder that misspellings abound in the literature. A common misspelling for the Atlantic sturgeon *Acipenser oxyrinchus* Mitchill, 1815 is *Acipenser oxyrhynchus*, and similarly for houting *Coregonus oxyrinchus* (Linnaeus, 1758) is *C. oxyrhynchus*. What all these species have in common is that they have something that could be called a pointed “nose”, which the original describers found so striking that they named their species for it.

Another issue is that spelling errors from the literature may be entered into databases, perhaps the error may occur during data entry, and then perpetuated, sometimes unknowingly, but on other occasions intentionally. For example the spelling error *Ammothea sextarticulata* (instead of *Ammothea sexarticulata* Munilla, 1990) was first published in 1994, and was entered as such in WoRMS in 2005. It was later corrected by the taxonomic editor but has already had 410 Google hits, whereas the correct spelling has had only 118 (checked 19 April 2012). In this case, the incorrect spelling is present in several online databases and continues to perpetuate in the literature; even the original author used the misspelling in 2008 [41]. To enable tracking of such errors, a taxonomic database should retain all published spellings but indicate which are in error.

### Economic consequences

The problems arising from incorrectly applying species names are not only of academic interest but have economic and conservation consequences. A species must have a scientific name to be included in the IUCN Red List which assesses the conservation status of species. Failure to correctly name pests and pathogens has resulted in wasted control measures [42], [43]. A major problem in tracking the status of fish populations is that catches are often mislabelled owing to reporters being unaware of related species and their correct names. FAO (UN Food and Agriculture Organisation) produced species identification guides so countries could better identify, and thus report, actual catches by species; instead of just listing ‘shark’ for example which could refer to any of hundreds of species. This correction resulted in an improvement from 46% to 95% of catch being reported at species level [44]. In Europe, five species of large skates have been landed under two species names, so the status of the stocks was unknown [45]. One species, the well-known European common skate, previously known as *Dipturus batis* (Linnaeus, 1758), became locally extinct in parts of Europe owing to overfishing but was recently proposed to consist of two previously described but synonymised species, *D. flossada* and the flapper skate, *D. intermedia*; the conservation status of both is now unclear [45]. The European sturgeon *Acipenser sturio* Linnaeus, 1758 is near extinction in Europe. It was assumed that it was the only sturgeon species in Europe, but examination of museum records found that sturgeons from the Baltic Sea, now extinct, were *A. oxyrinchus* which survives in NE America [46]. Thus, the species could be restocked to the Baltic. Many more cases of the importance of correct identification and naming of species are provided on the BioNET website.

### Biodiversity informatics

Several initiatives to better organise species names have been undertaken. In the early 1990s, van der Land [47] began to list species names through contacting experts and published the UNESCO-IOC Register of Marine Organisms (URMO) on diskette. In 1972 in the USA, NOAA's National Ocean Data Center developed a list of marine species names with code numbers, the NODC Taxonomic Code. This became part of the Integrated Taxonomic Information system (ITIS) in 1996 (http://www.nodc.noaa.gov/General/CDR-detdesc/taxonomic-v8.html). In 1997, Frank Bisby and colleagues launched a global effort called Species 2000 to link together and publish Global Species Databases (GSDs) on the internet, and subsequently also as the ‘Annual Checklist’ on CD-ROM. Most of the GSDs had not been previously published on the internet but sat on individual scientists' computers. The application of information technologies (IT) to biodiversity data, called biodiversity informatics, enables international collaboration and data management to be fast at low cost [48], [49], [50], [51], [52], [53].

The compilation of species names is aided by the ability of computers to search names from the literature and other databases [6]. Indeed, several important compilations of names exist, such as the Index of Organism Names (ION) which includes Zoological Record (www.organismnames.com) and the Global Names Index (http://gni.globalnames.org/). The latter now has 20 million name strings but this represents about 1.5 million accepted species when as yet unrecognised synonyms are accounted for [22]. ION has 1.5 million names and 1.2 million species and subspecies gathered from publications it regularly checks. Neither resource is revised by taxonomic experts so the validity of the names is not known. Gathering and classifying such names is essential, but finding the correct name to use for each species is more difficult. The same names may be used for an animal, plant or bacterium but because each of these groups is subject to different codes of nomenclature they are not considered homonyms.

Resolving taxonomic issues requires informed individuals who understand how the problems have arisen, know the rules and the literature well and have access to type specimens. The diversity of species limits the knowledge of any one expert to a particular taxon, sometimes with hundreds to thousands of species, and often only to the representatives of that taxon in a particular environment (e.g. marine) or geographic area. Thus, it takes many experts to cover all species, and some less popular or economically unimportant groups may have few or no experts. Species have been described in thousands of journals and books, so gathering the literature has also been expensive and time consuming. Here again the internet can help; for example by getting the old literature online, as underway by the Biodiversity Heritage Library. Not all species were well-described, especially those recognized early in the 19th century. Accurately applying species names often requires physical examination of the type specimens in a natural history museum or herbarium collection and their re-description. Knowing where these type specimens are located and accessing them is time-consuming and sometimes impossible. Thus Moretzsohn [54] proposed a special online database called TaxonBank, to register the location and other details of type specimens. The Australian Faunal Directory [55] includes type specimen information. Such a resource is needed for all species.

Scientific natural history museums and herbaria are depositories for reference collections of botanical, zoological, and paleontological specimens used in taxonomy and other life science disciplines. Synonymies are difficult to establish without reference to type specimens. These are kept in such collections and are accessible for that purpose [56]. Museum collections store specimens with collection data indicating locality and date of sampling. When there is uncertainty about species records if similar species are involved (including sibling species), then the study of museum specimens may yield solutions. This is also relevant when species have become locally extinct and past distribution ranges have to be reconstructed as for the Baltic Sea sturgeon [46]. Species that were believed to be endemic and became locally extinct would be considered globally extinct. However, they may be rediscovered in recent collections from elsewhere in the world indicating that they are still extant and that their status as endemics was erroneous [57]. The history of populations of non-native species reaching pest proportions in certain areas may also be traced back with the help of specimens deposited in museums. Comparisons of species lists of recent surveys and historical collections of the same areas, like in the proximity of large cities such as Jakarta or Singapore, may indicate that species have disappeared from their local faunas [58], [59], [60]. Thus these collections can be used to re-establish baselines in the context of historical ecology. They can also be increasingly important if they contain material from protected areas where species are not allowed to be sampled anymore [61]. It is the combined, complementary availability of marine biological collections worldwide that makes them useful for global change studies, which is enhanced as data pertaining to such specimens are made available in digital electronic form [56], [62]. Thus an online resource that indicates the location of specimens will aid researchers in correctly naming, identifying and classifying species; and improve quality control in taxonomy. For example, in the Swedish Museum of Natural History a Department of Biodiversity Informatics has been established which, amongst other things, will manage information about the collections.

Many authors have argued that the management and quality control of taxonomic and biodiversity data requires an online register of species [28], [63], [64], [65], [66], [67], [68]. However, there are practical limitations to what a group of scientists can achieve with limited resources. Providing a full web-based taxonomy, including expert-validated species nomenclatures and information on all species, is beyond the scope of a few scientists. However, clusters of scientists can contribute the parts of the ultimate resource, which is exactly what was achieved with the European Register of Marine Species (ERMS) [69], the Gulf of Mexico biodiversity inventory [70], AFD since the 1980s [55], and the New Zealand inventory of biodiversity [71], [72], [73]. In 1997–1999, ERMS was published on the internet and subsequently as a book [69]. This was notable in (a) bringing together over 170 experts to pool their knowledge on what species occurred in European seas into one database, (b) legally establishing the Society for the Management of Electronic Biodiversity Data (SMEBD) to hold the Intellectual Property Rights (IPR) of the contributors and thus facilitate the systems succession planning, and (c) having all the content in one standardised database [69], [74]. In 2000, the A. P. Sloan Foundation launched the Census of Marine Life (CoML), a decade of globally coordinated discovery in marine biology. CoML established an Ocean Biogeographic Information System (OBIS), which published species distribution data over the internet. This used a similar standard to, and is the largest marine contributor to the Global Biodiversity Information Facility (GBIF), established in 2001. These initiatives, and the increased use of databases to manage biological data, increased the demand for a standard checklist of marine species names and their relationships to synonyms. Following the completion of its start-up project, ERMS became hosted by a professional marine data centre at the Flanders Marine Institute (VLIZ). This provided the computing and data management infrastructure and support team on which to expand ERMS to become a World Register of Marine Species (WoRMS), and SMEBD provided the community of experts who invited colleagues to expand the content [51], [74], [75]. WoRMS was thus independent of, but collaborated with and contributed to, CoML and Species 2000. In this paper, we report how WoRMS has become an established part of the global biodiversity infrastructure, and is playing an increasingly important role in taxonomic data management.

## Methods

### Expert community

**Figure 1:**
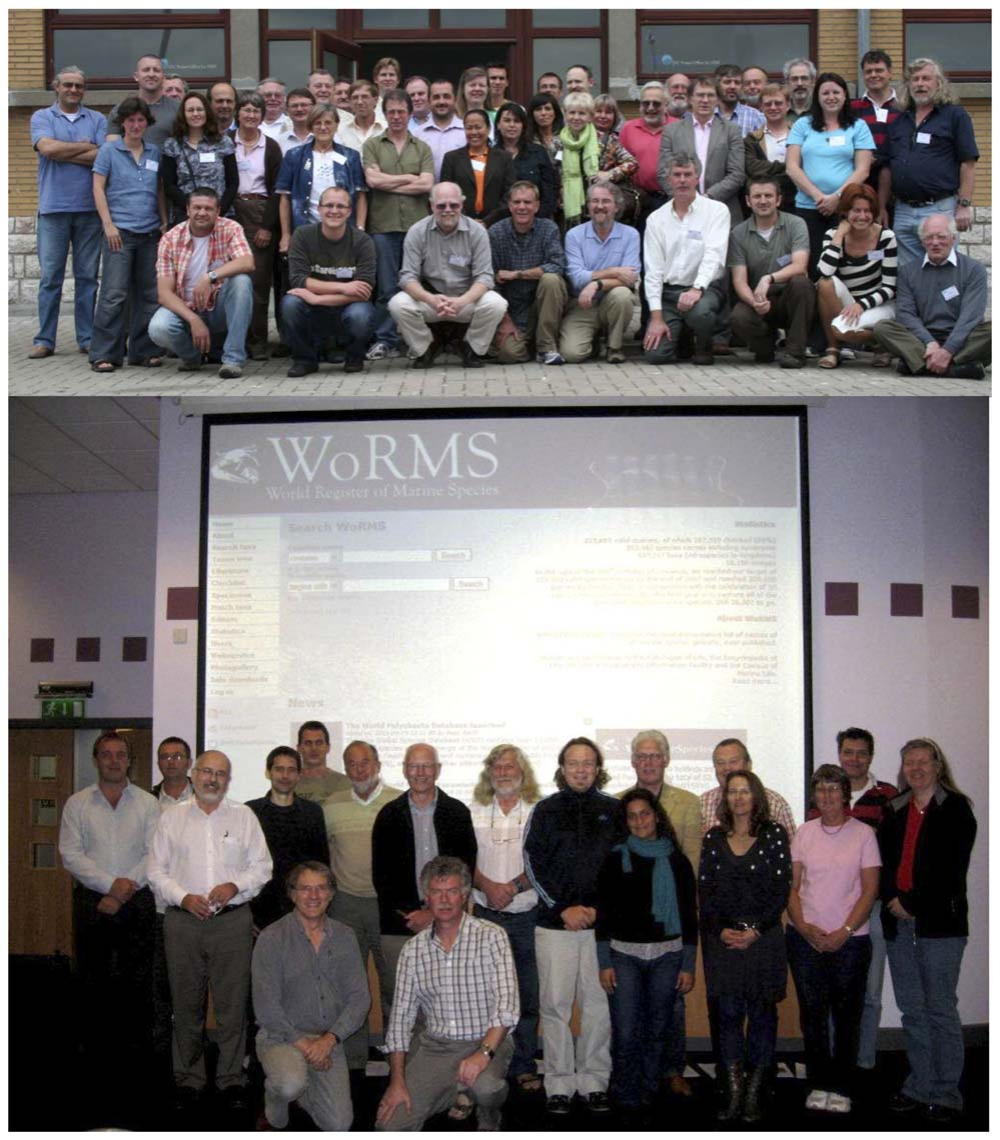
Some of the WoRMS editors at workshops in Ostend in 2008 (upper panel) and Aberdeen in 2011.

WoRMS editors were selected by their peers through knowledge of their publications and expertise in a taxon. The advantage of this approach was that the best known and most senior experts were first involved. They provided leadership and example to younger researchers and the wider community. However, the editors were encouraged to invite their colleagues to spread the workload and provide succession, including young researchers who may be more comfortable with using online databases for publication. Engaging potential editors was greatly helped by personal relationships and contacts at scientific meetings. In particular, the frequent workshops and meetings of the Census of Marine Life significantly helped such interactions, and most of the WoRMS Steering Committee (SC) members were involved in CoML. Two special WoRMS editors workshops have been held to determine policy and direction (Figure 1), but most coordination has been by email.

### Communication

The website is the primary method of communication. It includes News items which provide a history of WoRMS progress with links to further documents (e.g., reports of meetings), and Twitter feed with brief news items. Users can sign up to RSS feeds that will notify them of updates to WoRMS content. An email list provides the primary method for communication with editors.

### Host institution

Another important factor in attracting editors is the security a professional data centre provides for the continuity of the database. The data centre provides a database support team, so that changes in staff do not interrupt WoRMS development, and can provide 24/7 support, archiving, and professional IT system design and management. The host institution, VLIZ, is a leading Ocean Data Centre within the Intergovernmental Oceanographic Commission's (IOC) International Oceanographic Data and Information Exchange (IODE) programme, and a certified member of the World Data System of the International Council of Scientific Unions (ICSU). It finds the WoRMS database invaluable in its wider data-management activities. Thus it can support WoRMS as it supports other projects.

### Cost

The development of WoRMS, including ERMS since 1997, is estimated to have involved about €2 million in project funding for IT, editors' time, and meetings. However, the in-kind cost of SC members and editors' time directly involved in WoRMS is estimated at over €3 million. At present, the effort is equivalent to two full-time staff at the host-institution and similar in-kind effort by the editors, so including allowance for additional expenses, including overheads, a total annual cost of about €500,000 is estimated.

### Role of SMEBD

SMEBD was established to hold the Intellectual Property Rights of the ERMS, the precursor to WoRMS. The WoRMS SC was established within SMEBD to manage WoRMS. All contributors to WoRMS have the right to become honorary life-members of SMEBD. WoRMS editors nominate and elect people to the SC. As a legal entity and holder of the contributors' IPR, SMEBD has a key role in formally approving the host institution of the database, how it is disseminated, negotiating exceptional uses of the database, and following up on misuse of the data. For example, SMEBD successfully had a book withdrawn from publication because it had largely republished a WoRMS GSD without attribution of the source. SMEBD can also act as a contractor in research projects and manage their finances. It has been a full partner in two European Commission research contracts. SMEBD thus provides the governance for WoRMS. Its legal incorporation in Ireland requires it to have a detailed annual audit, and limits the financial liability of its Directors and members from any claims made against them in relation to the activities and assets of SMEBD. In contributing to the database, past and present, the editors have agreed to voluntarily provide data, information, opinion, or other expert assistance to the database. They retain the right to use and publish any data and intellectual property created by themselves, but authorise SMEBD to store, compile, modify, revise, and disseminate the data provided and derived by any means (e.g. electronic, World Wide Web, book). This includes appointing new editors who may add to and modify the original contributions of previous editors. They recognise that products of the database are the copyright of SMEBD, and they exercise control over the databases through election of the SMEBD Council. The WoRMS SC is elected from members nominated by its editors (SMEBD members).

### Content

The minimum requirement for WoRMS is an accepted full species name (i.e. accepted combination of genus, specific epithet, author, year) placed in an accepted higher taxon group (at least family) and environment (e.g. marine, brackish, terrestrial and/or freshwater). Desirable additional information is original genus-species combination (called basionym in plants), alternative past combinations, junior (subjective or otherwise) synonyms, key literature (ideally a link to the original publication), location of type material, and type locality. However, some species pages include considerable additional information, from biology to distributions and images. A system to label species fossil status and time stratigraphy is being added. Considerable data is entered by assistants, some at the host institution and others at editors' offices. This content is ‘quarantined’ until it is approved by the appropriate taxonomic editor.

### Citability

We recognised the importance of making the editors responsible for WoRMS visible on the web pages for two reasons. First, doing this indicates the authority behind the database content. Second, it was recognised that the editors wished their work to be recognised and attributed to them. We thus follow the well-established method of citing publications [76]. Each species and higher-taxon page has a citation at the foot of the page. Thus a user is expected to cite the species page, a higher-taxon page (e.g., Amphipoda), a GSD or the database as a whole, that is [77] depending on how they use it. WoRMS may be the first online biodiversity database to provide multiple levels of citation.

### Glossary

In preparation for the further expansion of the content to include ecological information, a glossary has been developed by a group of ecologists, geologists and taxonomists [78]. This is the first step to provide consistent definitions for use within WoRMS, i.e. a controlled vocabulary. This glossary is a collaboration between the scientists of the GEOHAB (Marine Geological and Biological Habitat Mapping) and WoRMS communities. It is authoritative in that definitions are approved by scientists who are well-established in the subject areas; peer-reviewed by both prior approval of experts and exposure to feedback from users; open-access (freely available online) for others to use; transparent by contributors and persons responsible being acknowledged; expert controlled by a small editorial group that approves changes to the definitions; and participatory in encouraging users to criticize definitions and suggest additional terminology for inclusion. The glossary can be expanded as users demand and experts are willing; modified based on feedback and changing use of terminology; contributes to data management by providing definitions for use of terminology in databases, and assists the development of ontologies that relate terms to each other. It is permanent with editors being replaced as their availability changes and new expertise is desirable; and contributes to associated initiatives including the Encyclopedia of Life (EoL), CoML, WoRMS, GBIF, OBIS, and IODE of IOC. It does not intend to provide a review or history of all uses of particular terms, nor how they may be used in other fields of research. However, a further development may be to make relationships between terms apparent in a ‘semantic ontology’. The definitions are those recommended for use in marine biology, ecology and geology. Where a term has different uses that the editors feel require clarification, these will be included. At present, this glossary excludes terminology specific to the following areas: names of marine species and higher taxa as these are in WoRMS; place names (see gazetteers at www.vliz.be/vmdcdata/vlimar and www.gebco.net/data_and_products/undersea_feature_names); taxonomy; physiology; archaeology; fisheries; legal and regulatory terms; and acronyms.

### Higher classification

The WoRMS editors determine the classification within the taxa for which they are responsible. However, the overall higher classification needed a standard approach to simplify data management. The first WoRMS editors' workshop discussed a proposal for a classification of Animalia to aid data management [79]. This, and the modification of all the other eukaryote kingdoms, has been adopted by the Species 2000 Catalogue of Life (CoL) [80], with a rationale explained by Gordon [81], and is implemented in WoRMS. The use of a common classification greatly aids data exchange. Its principles include: adoption following discussion with experts and consensus building; not implementing proposals for modifications to classification until there has been a year or two for them to be discussed by the taxonomic community; and only altering the classification at perhaps 5-year intervals. This conservative approach is designed to provide stability for data management, and so users do not get confused by new classifications and terminology.

### Infrastructure

The WoRMS data are stored in a relational MS SQL 2008 database called Aphia. An MS Access front end is built for administration purposes to control edit rights and perform quality control. The database contains over 440 fields, of which accepted species name is the most complete (100%). These fields are organised into 79 related tables described on the website at http://www.marinespecies.org/structure.

AphiaID provides a unique and permanent number for every species name within WoRMS (e.g. AphiaID 127160). It enables users to match up names in their databases with future versions of WoRMS, particularly where the status of a name may have changed (e.g. become a synonym) or the classification of the species may have changed. The AphiaID is included within the WoRMS LifeSience Identifier (LSID, http://sourceforge.net/projects/lsid), which is an implementation of a persistent Globally Unique Identifier (GUID). An example of a GUID is urn:lsid:marinespecies.org:taxname:127160. In addition, these LSIDs are resolvable and that they can produce structured taxonomic information in RDF (Resource Description Framework) format.

The editorial board has direct access to the database via a PHP (Hypertext Preprocessor) web interface. If editors prefer to work off-line they can use an MS Excel template, which is often also used for bulk updates. The WoRMS website is running on an Apache2 windows server, which backs up the data on a daily basis. The entire database is archived each month and users can download previous versions upon request. Copies of the database can be downloaded by organisations or individuals following approval by SMEBD. This involves completing a request form in which the recipient agrees not to further distribute the database or make it available online. These limitations are to avoid multiple or corrupted versions appearing on other websites, and to encourage users to contact WoRMS directly.

### Distribution maps

WoRMS stores published species distributions by using location names. The status of the location name (including different spellings and languages), coordinates, shapefiles, and geographic hierarchy is provided by linking to the VLIZ Marine Gazetteer (VLIMAR, http://www.vliz.be/vmdcdata/vlimar). The coordinates and shapefiles can be used to build species distribution maps, as currently implemented on the sponge database [82]. Maps are built using OpenLayers (www.openlayers.org), an open source javascript library to display dynamic maps in any web page. The back-end of both occurrence types is GeoServer (www.geoserver.org), an open source implementation of WMS that implements the Open Geospatial Consortium (OGC) standards.

### Photogallery

The WoRMS image library is a user-controlled facility for the upload and display of images adjusted for online publication (i.e. 800 px, 72–96 DPI) [83]. It is not necessary to create an account and log-in to do so, but account holders have edit privileges. It automatically resizes the image while storing the original size, to a 800 px wide ‘thumbnail’ image. If permitted, the original size can be provided online or made publicly available upon request. Video files can also be stored and displayed. The user must add some minimal metadata: including title, author, email, keywords; and terms of use (e.g. Creative Commons licence). It can automatically read embedded camera capture metadata (i.e. exif, gps) from uploaded pictures. The keywords are part of a controlled vocabulary and multiple entries are possible. A drop-down list of taxonomic names avoids users entering misspellings.

Editors can link images to specimens, which can have additional metadata (e.g. details on code number, storage, identification, locality, biology etc). Because many images are not uploaded by the taxonomic editors, the species they contain may not be correctly identified or the image may not be of sufficient quality for species recognition. Thus whether or not the image has been verified by an editor is indicated.

Images can then be searched on species name, title, author and other keywords. There is an option to allow users to provide comments, which are moderated by the database administrator. Because they are associated with species names, the images are thus available to all Regional Species Databases in WoRMS, and can be accessed by external organisations, such as the Encyclopedia of Life. The number of times an image has been viewed is tracked.

## Results

### Content

**Figure 2:**
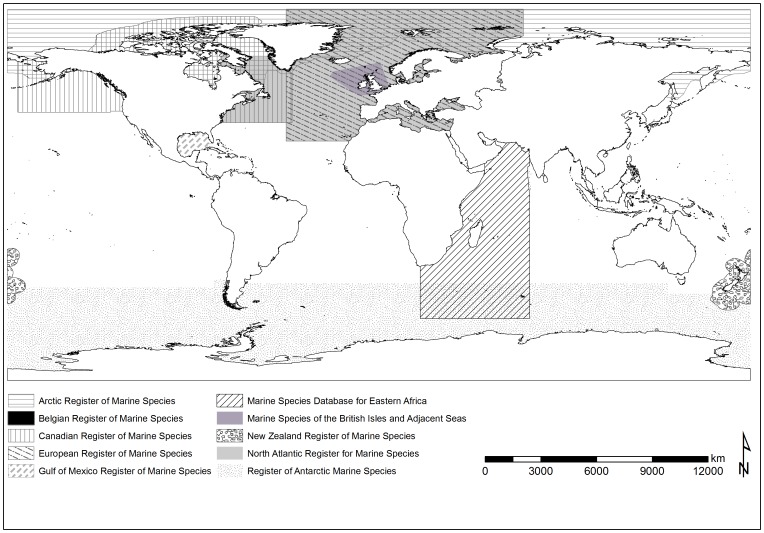
The approximate present geographic coverage of the larger Regional Species Databases (i.e. all-species inventories) within WoRMS.

In 2012, WoRMS contained almost 100 global, 12 regional and 4 thematic species databases overseen by 240 editors (Tables 2, 3). The editors are located in 133 institutions and 31 countries (Table 4). Of the GSDs, 22 have their own entry web page which provides scope for the editors to provide additional background and profile for their taxon (Table 2). Regional Species Databases (RSDs) cover less than half the oceans (Figure 2), but additional RSDs are planned. The RSD editors add distributional context to WoRMS, and work with the GSDs taxonomic editors to resolve nomenclatural discrepancies and omissions. Some editors are involved in a GSD, RSD and/or Thematic Species Database (TSD).

**Table 2:** The Global Species Databases hosted within WoRMS. Those with their own web entry page are underlined.

Acarina marine: Bartsch, I.	Holothuroidea: Paulay, G.
Achelata: Chan, T-Y., Fransen, C.H.J.M.	Hydrozoa: Schuchert, P.
Actiniaria: Fautin, D.	Insecta marine: Cheng, L.
Alcyonacea: van Ofwegen, L.P.	Isopoda marine, freshwater and terrestrial: Schotte, M., Boyko, C.B, Bruce, N.L., Poore, G.C.B., Taiti, S., Wilson, G.D.F.
Amphipoda: Lowry, J.	Larvacea: Hopcroft, R.
Antipatharia: Molodtsova, T., Opresko, D.	Leptostraca: Mees, J., Walker-Smith, G.
Ascidiacea :Shenkar, N., Gittenberger, A., Lambert, G., Rius, M., Moreira Da Rocha, R., Swalla, B.J., Turron, X.	Lithodoidea: Ahyong, S.
Ascothoracida: Grygier, M.J.	Lophogastrida, Stygiomysida and Mysida: Mees J., Meland K.
Aspidogastrea: Cribb, T.	Mangroves: Dahdouh-Guebas F.
Astacidea: Chan, T-Y.	Merostomata: Boxshall, G.
Asteroidea: Mah, C.L.	Monogenea: Gibson D., Bray R.
Axiidea: Poore, G.	Monoplacophora: Bouchet P., Gofas S., Rosenberg G.,
Bivalvia: Bouchet, P., Gofas, S., Rosenberg, G.	Myriapoda littoral: Barber, A.D.
Bochusacea: Boxshall, G.A.	Mystacocarida: Boxshall, G.A.
Brachiopoda: Emig C.C., Alvarez F., Bitner M.A.	Myxozoa: Karlsbakk, E., Adlard, R.
Brachypoda: Boxshall, G.A.	Nematomorpha: Neuhaus, B., Schmidt-Rhaesa, A.
Brachyura marine: Ng, P.K.L., Davie, P.	Nemertea: Norenburg J., Gibson R.
Branchiopoda marine & brackish: Boxshall, G.	Oligochaeta marine: Timm, T., Erséus, C.
Branchiura: Boxshall, G., Walter, T. C.	Ophiuroidea: Stöhr, S., O'Hara, T.
Bryozoa: Bock, Phil, Gordon, D.	Orthonectida and Rhombozoa: Furuya, H., Hochberg, E.
Caridea: De Grave, S., Fransen, C H.J.M.	Paguroidea & Lomisoidea: Lemaitre, R., McLaughlin, P.
Carnivora marine: Berta, A., Churchill, M.	Pennatulacea: Williams, G.
Caudofoveata: Bouchet, P., Gofas, S., Rosenberg, G.,	Pentastomida: Boxshall, G.
Cephalopoda: Bouchet, P., Gofas, S., Rosenberg, G.,	Phoronida: Emig, C.C.
Ceriantharia: Molodtsova, T.	Placozoa: Schierwater, B., Eitel, M., DeSalle, R.
Cetacea: Perrin, W.F.	Podocopa: Nunes Brandao, S.
Chaetognatha: Thuesen, E.V., Pierrot-Bults, A.	Polychaeta: Read, G., Fauchald, K.
Chirostyloidea & Galatheoidea: Macpherson E., Schnabel K.	Polychelida: Chan, T-Y., Ahyong, S.
Ciliophora: Warren, A., Agatha S, Dolan J	Polycystina (Radiolaria): Lazarus, D.
Cirripedia: Chan, Benny K.K, Boxshall, G.	Polyplacophora: Schwabe, E.
Copepoda: Walter, T.C., Boxshall, G.	Porifera: Van Soest R.W.M, Boury-Esnault N., Hooper, J.N.A., Rützler, K, de Voogd, N.J., Alvarez de Glasby, B., Hajdu, E., Pisera, A.B., Manconi, R., Schoenberg, C., Janussen, D., Tabachnick, K.R., Klautau, M., Picton, B., Kelly, M., Vacelet, J.
Corallimorpharia: Fautin, D.	Priapulida: Neuhaus, B., van der Land, J.
Crinoidea: Messing, C.	Proseriata and Kalyptorhynchia - Rhabditophora: Artois T., Schockaert E., Tyler S.
Cumacea: Watling, L., Gerken, S.	Pycnogonida: Bamber, R.N., El Nagar, A.
Dendrobranchiata: De Grave, S., Fransen, C.	Remipedia: Koenemann, S., Hoenemann, M., Stemme T.
Digenea marine: Cribb, T., Gibson, D.	Reptilia marine: Uetz, P., Hallermann, J.
Echinoidea: Kroh, A. & Mooi, R.	Scaphopoda: Scarabino, V.
Echiura: Murina, V.	Scleractinia: Cairns, S.D., Hoeksema, B.W.
Entoprocta: Iseto, T., Nielsen, C.	Scyphozoa: Jarms, G., Lindsay, D.
Euphausiacea: Siegel, V.	Seabirds: Adriaens, P.
Facetotecta: Boxshall, G.	Sipuncula: Saiz, J.
Foraminifera modern: Hayward B.W., Cedhagen T., Kaminski M., Gross O.	Sirenia marine: Self-Sullivan, C.
Gastropoda: Bouchet P., Gofas S., Rosenberg G.	Solenogastres: Garcia-Alvarez, O.
Gastrotricha: Todaro, A., d'Hondt, J-L.	Staurozoa: Collins, A.G., Mills, C.
Gebiidea: Poore, G.	Stenopodidea: De Grave, S., Fransen, C H.J.M.
Gnathostomulida: Sterrer, W.	Tanaidacea: Anderson, G., Blazewicz, M.
Halocyprida: Angel, M.	Tantulocarida: Boxshall, G.A.
Helioporacea: van Ofwegen, L.P.	Thermosbaenacea: Poore, G.
Hemichordata: Shenkar, N, Swalla, B.J., van der Land, J.	Xenoturbellida: Gofas, S.
Hippoidea: Boyko, C.	Zoanthidea: Reimer, J.D., Sinnger F.
Hirudinea marine: Kolb, J.	

**Table 3:** The (a) Regional Species Databases (RSD) and (b) Thematic Species Databases (TSD), hosted within WoRMS, and their editors.

(a) RSD
**European Register of Marine Species (ERMS):**
Costello, M.J.; Bouchet, P.; Boxshall, G.; Arvantidis, C.; Appeltans, W.
**Canadian Register of Marine Species (CaRMS):**
Kennedy, M.K., L. Van Guelpen, G. Pohle, L. Bajona
**The SCAR-MarBIN Register of Antarctic Marine Species (RAMS):**
De Broyer, C.; Clarke, A.; Koubbi, P.; Pakhomov, E.; Scott, F.; Vanden Berghe, E.; Danis, B.
**MArine Species Database for Eastern Africa (MASDEA):**
Vanden Berghe, E.
**Black Sea checklist for Ocean-Ukraine & Sibema:**
Sergeyeva, O.
**The New Zealand Inventory of Biodiversity:**
Gordon, D.
**The Belgian Register of Marine Species (BeRMS):**
VLIZ Belgian Marine Species Consortium
**Gulf of Mexico Register of marine species:**
Tunnel, W.; Moretzsohn, F.
**The Arctic Register of Marine Species (ARMS) compiled by the Arctic Ocean Diversity (ArcOD):**
Sirenko, B.I.; Clarke, C.; Hopcroft, R.R.; Huettmann, F.; Bluhm, B.A.; Gradinger, R.
**Marine Species of the British Isles and Adjacent Seas (MSBIAS):**
The UK Marine Environmental Data and Information Network
**North Western Atlantic Marine Species Register (NWARMS):**
Forster, S.; Van Guelpen, L.; Pohle, G.; Martin, A.; Welshman, D.
**African Register of Marine Species:**
Odido, M.; Appeltans, W.; Bel Hassen, M.A.; Jiddou, A.M.; Mussai, P.; Nsiangango, S.E.; Vandepitte, L.; Wambiji, N.; Zamouri, N.
(b) TSD
**North Sea Benthos Project:**
Rees, H.; Cochrane, S.J.; Craeymeersch, J.A.; de Kluijver, M.; Degraer, S.; Desroy, N.; Dewarumez, J.-M.; Duineveld, G.; Essink, K.; Hillewaert, H.; Kilbride, R.; Kröncke, I.; Nehmer, P.; Rachor, E.; Reiss, H.; Robertson, M.; Rumohr, H.; Vanden Berghe, E.
**Northsea Benthos Survey:**
Craeymeersh J., P. Kingston, E. Rachor, G. Duineveld, C. Heip., E. Vanden Berghe. (1986).
**IOC-UNESCO Taxonomic Reference List of Harmful Micro Algae:**
Moestrup, Ø., Akselman, R., Cronberg, G., Elbraechter, M., Fraga, S., Halim, Y., Hansen, G., Hoppenrath, M., Larsen, J., Lundholm, N., Nguyen, L. N., Zingone, A.
**UNESCO-IOC Register of Marine Organisms (URMO):**
Land J. van der

**Table 4:** The countries and institutes represented by the editors of WoRMS and its associated databases. These are mapped at http://www.marinespecies.org/imis.php?module=gmap&spcolid=507.

**Argentina:** Instituto Nacional de Investigación y Desarrollo Pesquero; Universidad Nacional de Mar del Plata.
**Australia:** Australian Museum; Australian Antarctic Division; Australian Institute of Marine Science; Ecologia Environment; Macquarie University; Museum Victoria, Melbourne; Natural Sciences Museum & Art Gallery of the Northern Territory; Queensland Museum; South Australian Museum; Tasmanian Museum and Art Gallery; University of Queensland; University of Tasmania; University of Western Australia.
**Austria:** Natural History Museum Vienna; University of Innsbruck; Universität Salzburg; University of Vienna.
**Belgium:** Koninklijk Belgisch Instituut voor Natuurwetenschappen; Royal Belgian Institute of Natural Sciences; Université Libre de Bruxelles; Universiteit Gent; Universiteit Hasselt; Vlaams Instituut voor de Zee; Vlaamse Overheid; Beleidsdomein Leefmilieu, Natuur en Energie; Instituut voor Natuur- en Bosonderzoek; Afdeling Biodiversiteit en Natuurlijk Milieu; Vrije Universiteit Brussel.
**Bermuda:** Natural History Museum.
**Brasil:** Universidade Federal do Paraná; Universidade Federal do Rio de Janeiro.
**Brunei**: University Brunei Darussalam.
**Canada:** University of British Columbia; Fisheries & Oceans Canada, Bedford Institute of Oceanography; The Atlantic Reference Centre of the Huntsman Marine Science Centre.
**Denmark:** Natural History Museum; University of Aarhus; University of Copenhagen.
**Estonia:** Estonian University of Life Sciences.
**France:** Association Française de Conchyliologie; BrachNet; Centre National de la Recherche Scientifique and Université de la Méditerrannée; Centre d'Océanologie de Marseille; Muséum National d'Histoire Naturelle; Université de Bourgogne; Université des Sciences et Technologies de Lille; Université Pierre & Marie Curie Paris 6; Station Marine de Wimereux.
**Germany:** Alfred Wegener Institute for Polar- and Marine Research; Bavarian State Collection of Zoology; Christian-Albrechts- Federal Research Centre for Fisheries; Johann-Heinrich-von-Thuenen Institut; Leibniz Institute of Marine Sciences; Museum für naturkunde; School of veterinary medicine Hannover; Senckenberg Nature Research Society; Senckenbergische Naturforschende Gesellschaft; Senckenberg Naturmuseen und Forschungsinstitute; University of Hamburg; University Kiel; University of Siegen; Ludwig Maximilians University Munich; Zoological Institute und Zoological Museum;.
**Greece:** Aristotle University of Thessaloniki.
**Hong Kong:** University of Hong Kong.
**Ireland:** National University of Ireland (Galway); Ulster Museum.
**Israel:** Tel-Aviv University.
**Italy:** Consiglio Nazionale delle Ricerche; Italian National Research Council; Stazione Zoologica ‘Anton Dohrn’ di Napoli; Università degli Studi di Genova; University of Lecce; Università degli studi di SASSARI; University of Salento; University of Modena e Reggio Emilia; University of Rome La Sapienza.
**Japan:** Japan Agency for Marine-Earth Science and Technology; Kyoto University; Seto Marine Biological Laboratory; Osaka University; Shimane University; Toho University; University of Ryukyus.
**Mexico:** El Colegio de la Frontera Sur, Unidad Chetumal.
**Netherlands:** HAS Den Bosch; Netherlands Centre for Biodiversity Naturalis; Universiteit Leiden; Universiteit van Amsterdam.
**New Zealand:** Geomarine Research; Massey University; National Institute of Water and Atmospheric Research; University of Auckland.
**Norway:** University of Bergen; University of Tromso.
**Philippines:** Worldfish Center.
**Poland:** Polish Academy of Sciences; University of Lodz.
**Romania:** Muzeul National de Istorie Naturala Grigore Antipa.
**Russia:** Moscow State University; Russian Academy of Sciences; A.N. Severtsov Institute of Ecology and Evolution; P. P. Shirshov Institute of Oceanology; Pacific Institute of Geography; Zoological Institute.
**Saudi-Arabia:** King Fahd University of Petroleum and Minerals;
**Singapore:** National University of Singapore.
**South-Africa:** University of Pretoria.
**Spain:** Consejo Superior de Investigaciones Científicas; Insituto Español de Oceanografia; Universitat Autonoma de Barcelona; Universitat de les Illes Balears; Universidad de Sevilla; University of Málaga; University of Oviedo; University of Santiago de Compostela; University of the Basque country; University of Valencia.
**Sweden:** Swedish Museum of Natural History; University of Gothenburg.
**Switzerland:** Natural History Museum of the city of Geneva; University of Zurich.
**Taiwan** [Ta-Chunghwa]: Academia Sinica; National Taiwan Ocean University.
**UK:** ARTOO Marine Biology Consultants; British Antarctic Survey; British Myriapod and Isopod Group; Cab International; Centre for Environment, Fisheries and Aquaculture Science; Weymouth Laboratory; International Commission for Zoological Nomenclature; Joint Nature Conservation Committee; National Oceanography Centre, Southampton; Natural History Museum; University of Cambridge; University of Oxford; University of Southampton;
**Ukraine:** Kharkiv National University.
**USA:** Academy of Natural Sciences; Agnes Scott College; American Museum of Natural History; Brigham Young University; California Academy of Sciences; California State University; Dowling College; Federal Government of the United States of America; The National Systematics Laboratory; Field Museum of Natural History; George Washington University; Harte Research Institute for Gulf of Mexico Studies; Illinois Natural History Survey; Marine Biological Laboratory; National Oceanic and Atmospheric Administration; Fisheries Service; Southwest Fisheries Science Center; Natural History Museum of Los Angeles County; Natural Sciences, Museum & Art Gallery Northern Territory; Nova Southeastern University; Oak Ridge National Laboratory; Ohio University;San Diego State University; San Francisco State University; Santa Barbara Museum of Natural History; Sirenian International, Inc; Smithsonian Institution; National Museum of Natural History; The Evergreen State College; The University of Southern Mississippi; University of Alaska Anchorage; University of Alaska Fairbanks; University of California, Davis; University of California, Merced; University of California, San Diego; Scripps Institution of Oceanography; University of California, Santa Cruz; University of Florida; Florida Museum of Natural History; University of Kansas; University of Maine; University of Washington; University of Wyoming; Virginia Commonwealth University; Woods Hole Oceanographic Institution.
**Venezuela:** Instituto Venezolano de Investigaciones Científicas.

One principle in setting up WoRMS was to not ask taxonomists to repeat their work. Thus WoRMS built on authoritative registers of all-taxon marine species lists that existed at regional levels (e.g. Europe) and for particular taxa at global levels. Several GSDs were incorporated into WoRMS, including the world databases on all marine, freshwater and terrestrial Copepoda and Isopoda developed at the Smithsonian Institution, and world databases on Cumacea, Brachiopoda and Phoronida. Externally sourced content is from the collaborating databases: Biogeoinformatics of the Hexacorals (sea anemones and their relatives), World list of marine Fungi (from Index Fungorum), World list of Marine Pisces (from Catalog of Fishes via FishBase), World list of Algae (from AlgaeBase) [84], World list of free-living Nematodes – NeMys, World list of Marine Rotifers (from FADA), World list of marine reptiles (From Reptile Database), World list of Turbellaria (from Turbellarian Taxonomic Database), World list of Recent and Fossil Bryozoa, and the World list of Ctenophora. WoRMS is updated by content from these scholarly resources, and, in turn WoRMS provides its content and/or services to other resources that might otherwise need to recreate it.

**Figure 3:**
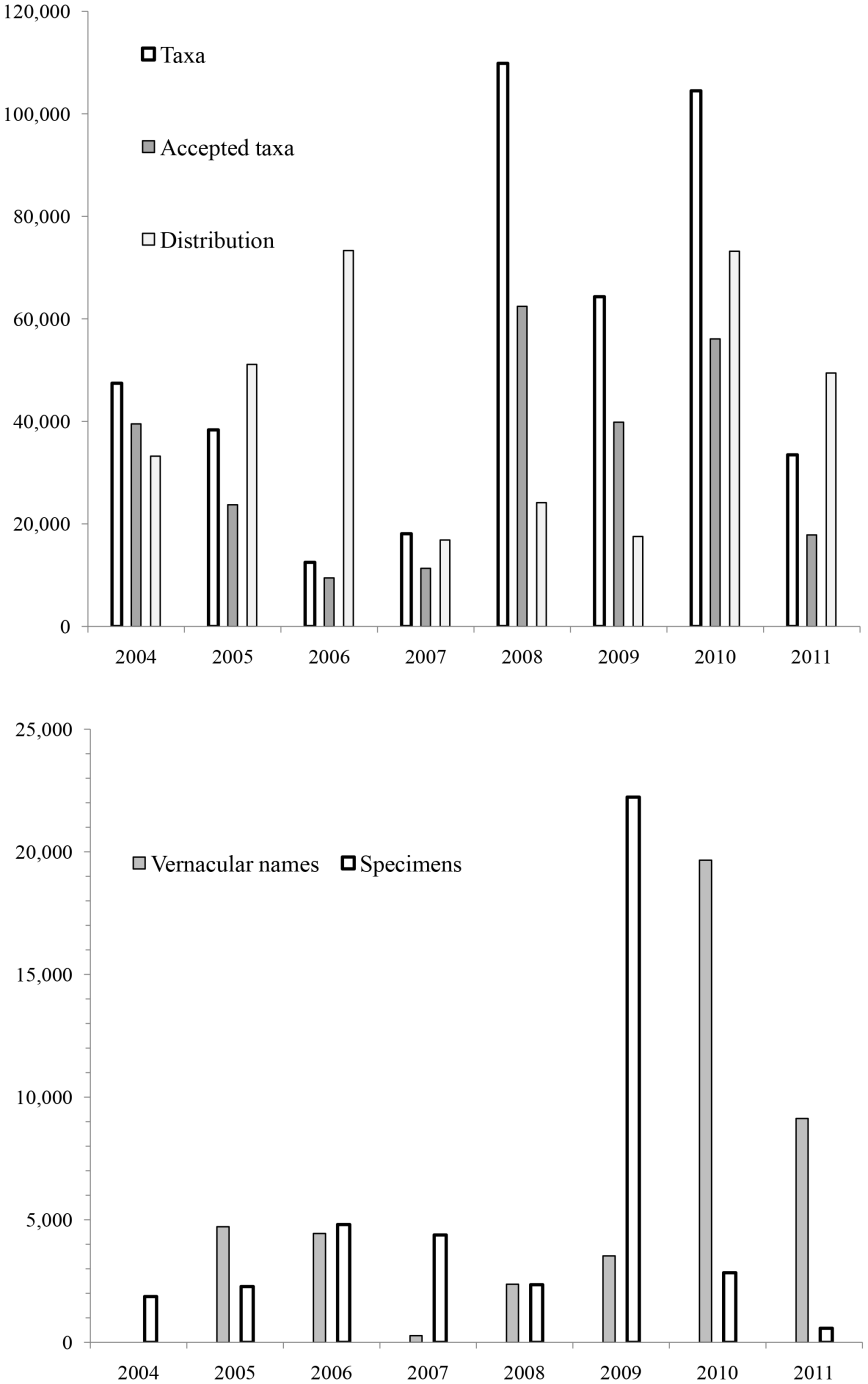
Annual numbers of taxa names (species and above including synonyms), accepted species names, vernacular names, distribution data, and specimens, added to WoRMS and its precursor ERMS.

At present, WoRMS contains 460,000 taxonomic names (from kingdom to species), and 368,000 species names. The latter include synonyms, nomina dubia, nomen nuda, misspellings, and old genus combinations. The species with the most synonyms is the breadcrumb sponge *Halichondria panicea* (Pallas, 1766), with 64. There are 215,000 accepted species names (Table 5). About 10% of the species names, entered by data assistants or editors, remain to be checked by editors. Some editors make their taxon complete across all environments, so there are 26,000 non-marine species also in the database (Table 5). Associated information includes about 150,000 literature sources, 20,000 pictures, and information on 44,000 specimens, of which over 5,000 are holotypes. Specimen information in museum collections can be matched to species. For example, WoRMS has over 40,000 linked taxon records to the invertebrates deposited at the Smithsonian Institution, National Museum of Natural History. ERMS was moved to the present host institution in 2004 and once WoRMS was launched in 2008 significantly more content was entered (Figure 3). Since 2010 there have been fewer additional species to enter and thus effort has shifted to other content, notably vernacular names and distribution data (Figure 3).

**Table 5:** The number of marine (a) taxa (infra-species and above), (b) species names (includes synonyms), (c) accepted species names (excluding synonyms); and (d) additional non-marine species in the WoRMS database (note that there are additional non-marine species of many taxa not in WoRMS).

(A) Eukaryota	(a) all taxa	(b) all species	(c) accepted	(d) non-marine
Biota	461,023	368,426	215,016	26,481
ANIMALIA	389,632	320,860	183,987	16,997
Acanthocephala	621	485	411	0
Annelida	24,598	20,703	12,751	20
Arthropoda	95,749	78,309	55,425	11,817
Brachiopoda	673	450	390	0
Bryozoa	8,048	6,481	5,990	0
Cephalorhyncha	274	213	211	0
Chaetognatha	342	281	129	0
Chordata	69,589	56,290	21,789	515
Cnidaria	17,618	14,785	10,873	6
Ctenophora	354	229	187	0
Cycliophora	6	2	2	0
Echinodermata	19,669	16,039	7,105	7
Echiura	296	234	198	0
Entoprocta	226	207	172	0
Gastrotricha	622	523	451	387
Gnathostomulida	151	109	97	0
Hemichordata	267	148	125	0
Mesozoa	195	165	134	0
Mollusca	97,901	82,558	41,642	108
Myxozoa	637	563	473	0
Nematoda	9,364	7,879	6,935	10
Nemertea	3,253	2,669	1,360	0
Phoronida	35	31	16	0
Placozoa	6	3	1	0
Platyhelminthes	15,503	11,959	8,050	2,764
Porifera	20,262	17,057	8,143	449
Rotifera	334	208	186	40
Sipuncula	1,645	1,283	148	0
Tardigrada	472	331	170	867
Xenacoelomorpha	858	666	423	7
FUNGI	2,680	1,545	1,209	57
Ascomycota	2,055	1,254	954	25
Basidiomycota	199	58	56	28
Chytridiomycota	88	47	38	1
Microsporidia	249	165	140	2
PLANTAE	24,457	16,907	9,028	6,149
Chlorophyta	4,650	2,575	1,960	1,985
Glaucophyta	11	0	0	18
Plantae incertae sedis	287	4	2	2
Rhodophyta	16,939	13,217	6,315	558
Streptophyta	2,492	1070	728	3,585
FUNGI	2,680	1,545	1,209	57
Ascomycota	2,055	1,254	954	25
Basidiomycota	199	58	56	28
Chytridiomycota	88	47	38	1
Microsporidia	249	165	140	2
Zygomycota	38	16	16	0
PROTOZOA	1,658	707	574	38
Amoebozoa	263	132	113	0
Apusozoa	14	3	0	0
Choanozoa	315	210	159	2
Euglenozoa	440	296	240	32
Loukozoa	11	2	2	0
Metamonada	54	17	14	0
Percolozoa	38	15	13	0
CHROMISTA	37,707	26,351	18,350	3,050
Bigyra	182	101	80	0
Cercozoa	361	189	165	0
Ciliophora	3,845	2,912	2,653	1
Cryptophyta	208	128	89	17
Foraminifera	11,025	8,578	6,467	1
Haptophyta	741	371	266	5
Heliozoa	24	10	10	0
Myzozoa	5,146	3,895	2,764	137
Ochrophyta	15,075	9,530	5,385	2,889
Oomycota	126	63	44	0
Radiozoa	934	574	427	0

(B) Prokaryota	(a) all taxa	(b) all species	(c) accepted	(d) non-marine
BACTERIA	4,159	1,939	1,751	190
Acidobacteria	9	1	1	0
Actinobacteria	264	71	71	0
Aquificae	28	15	15	0
Bacteria incertae sedis	30	1	1	0
Bacteroidetes	424	235	235	0
Caldiserica	5	1	1	0
Chlamydiae	9	0	0	0
Chlorobi	12	4	4	0
Chloroflexi	34	3	3	0
Cyanobacteria	1,349	688	505	190
Deferribacteres	11	4	4	0
Deinococcus-Thermus	17	4	4	0
Elusimicrobia	4	0	0	0
Fibrobacteres	4	0	0	0
Firmicutes	293	94	94	0
Fusobacteria	15	4	4	0
Gemmatimonadetes	4	0	0	0
Lentisphaerae	4	1	1	0
Nitrospirae	5	0	0	0
Planctomycetes	17	4	4	0
Proteobacteria	1,477	763	758	0
Spirochaetes	10	1	1	0
Synergistetes	12	3	3	0
Tenericutes	19	3	3	0
Thermodesulfobacteria	7	2	2	0
Thermotogae	22	12	12	0
Verrucomicrobia	46	25	25	0
ARCHAEA	232	117	117	0
Crenarchaeota	48	19	19	0
Euryarchaeota	171	96	96	0
Thaumarchaeota	9	2	2	0
VIRUSES	459	0	0	0

Kingdom names are capitalised. (1) includes Tracheophyta and Marchantiophyta. (2) includes Sarcomastigota.

### Matching taxa

Determining the correct spelling of a scientific name is not always a trivial task (e.g., which one is correct: *Cirrhitichthys*, *Cirrhitychthys* or *Cirritichthys?*) and it is very difficult for non-taxonomists to keep up with the status of species names. WoRMS has an online, semi-automated name validation tool called Taxon Match, to cross-check the spelling and taxonomic status of species against the WoRMS database. The tool is an implementation of the TaxaMatch algorithm which comprises a suite of custom filters and tests used in succession on genus, species epithet, plus authority where supplied [85]. It also uses the Scientific Names Parser [86]. The tool returns standard taxonomic information in a user-friendly format (e.g., MS Excel or tab delimited text file). The user needs to upload a list of species names, match the columns with the fields in the database and the system will return the file with valid names. The tool corrects the spelling if there are close matches found, notifies when the name is an unaccepted synonym, and provides the authority and publication date, the hierarchical classification, quality status (expert validated or not), and the WoRMS LSID. Up to 95% of common spelling mistakes are captured. When there are multiple potential matches the system provides a pick-list. It is a very popular tool, already appreciated by thousands of users (with on average 14 files uploaded on a daily basis).

### Web services

In contrast to the Taxon Match, where the user has to upload a species list, the portal also provides a platform-independent web service, that is it can run on PC, Mac and Linux operating systems. It uses the Web Services Description Language (WSDL) and Simple Object Access Protocol (SOAP) to enable data exchange. This web service allows users to dynamically link their own applications to the WoRMS database and will allow them to match a locally stored species list and add taxonomic and additional information derived from WoRMS (Table 6). WoRMS is also linking with other online data systems (link-out). They may use the web-services and/or use the WoRMS Taxon Match tool to cross-link names in their database andlink back to WoRMS (link-in) (Table 7).

**Table 6:** Examples of possible applications of the WoRMS web service.

Function	Parameter
getAphiaID	Scientific Name
getAphiaRecords	Scientific Name
getAphiaNameByID	AphiaID
getAphiaRecordByID	AphiaID
getAphiaRecordByTSN	TSN
getAphiaRecordsByNames	Scientific Name
getAphiaRecordsByVernacular	Common name
getAphiaClassificationByID	AphiaID
getSourcesByAphiaID	AphiaID
getAphiaSynonymsByID	AphiaID
getAphiaVernacularsByID	AphiaID
getAphiaChildrenByID	AphiaID
metadata()	LSID

**Table 7:** Examples of how WoRMS links with other online biodiversity resources.

Link-out	System	Link-in	Mechanism	Content applications
ID	EoL	AphiaID	web service	taxonomy, distributions, sources, notes, citations
ID	NCBI taxon	AphiaID	LinkOut	taxon name
ID	BoLD	/	web service	taxon name
/		AphiaID	taxamatch	taxon name, parent taxon, child taxa, synonyms, attributes
ID	IUCN Red List	AphiaID	web service	taxon name, conservation status
ID	ITIS	/	file transfer	
/	WikiSpecies NL	AphiaID	file transfer	Taxonomy
/	Wikipedia	AphiaID	web service	Taxonomy
name	BHL	/	web service	bibliographic metrics, #papers, pages
/	OBIS	AphiaID	taxamatch	Taxonomy
/	CoL	AphiaID	file transfer	taxonomy, distributions, citations
ID	VLIMAR	AphiaID		regional checklists, hierarchical search, latitude & longitude of place names
ID	Plankton-Net	AphiaID	taxamatch	taxon name, picture sharing
ID	IMIS	AphiaID	taxamatch	metadata, Ref/person/inst
ID	BR Meise	AphiaID	taxamatch	Specimen image, zoom

### Usage

**Figure 4:**
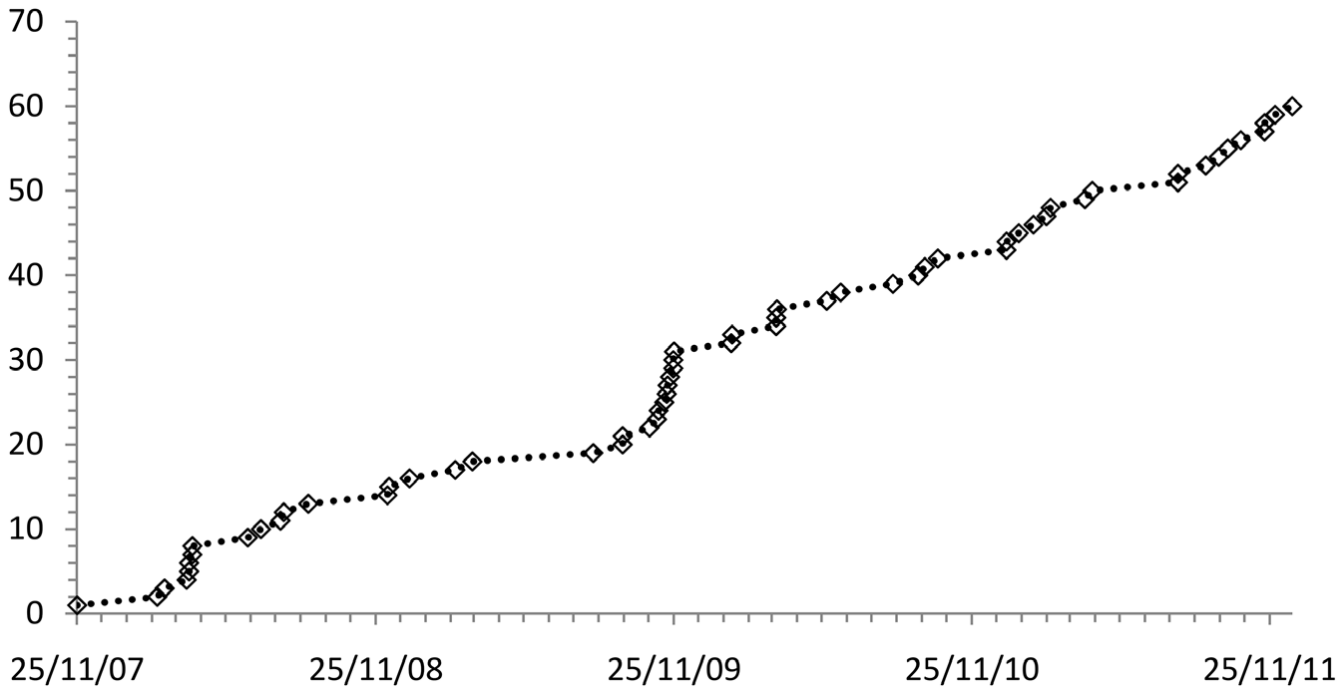
The number of organisations using WoRMS for their research and/or data management as listed in **Table 9**.

**Figure 5:**
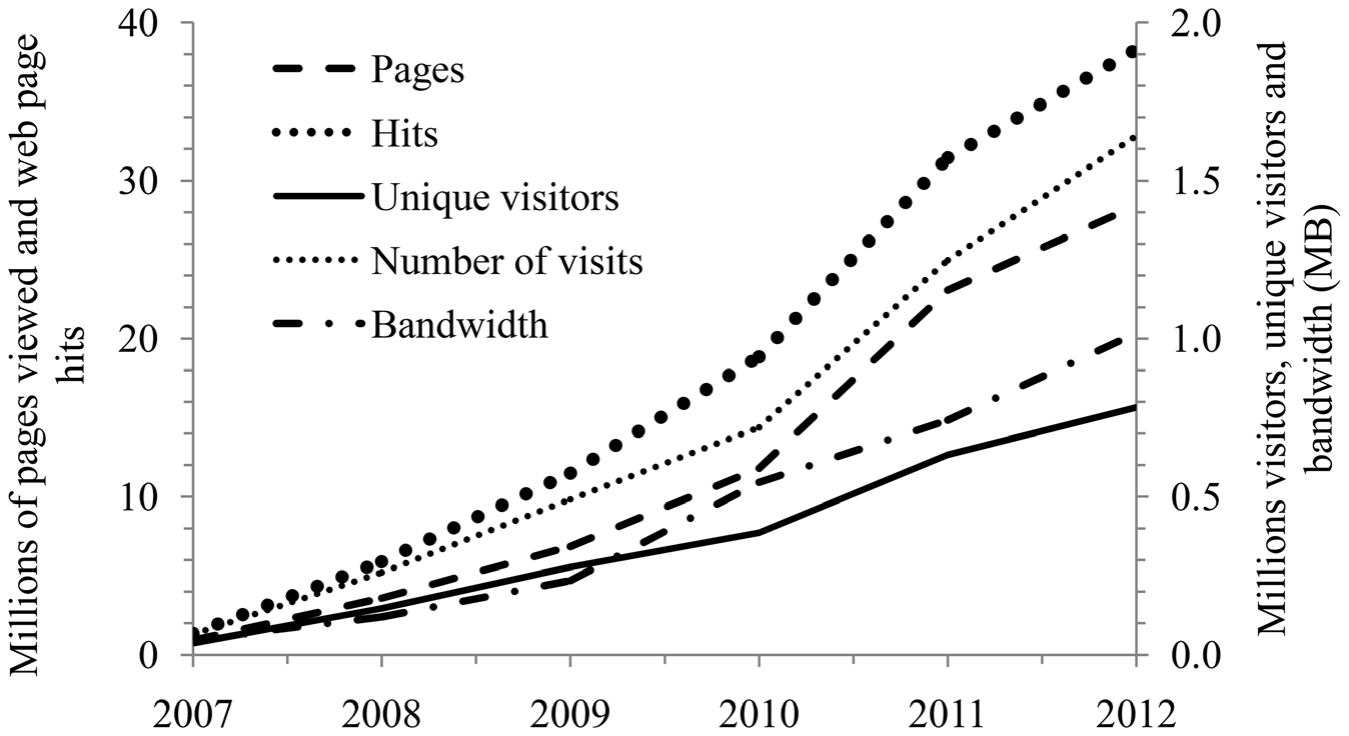
The annual use of the WoRMS website since January 2007 in terms of pages viewed and web page hits (left axis), and numbers of visits, unique visitors and bandwidth (MB) (right axis). The data for 2012 are estimated based on the trend until October.

The WoRMS web service is being used by at least 28 organisations from 12 countries (Table 8). Permission is not required to use the Web service so there may be additional users we are not aware of. Copies of the database have been licensed out to 61 organisations in 21 countries (Table 9) with demand growing steadily (Figure 4). Since 2007, all website-use metrics show a steady increase in access (Figure 5). There were about 600,000 unique visitors in 2011, and 57,000 in December 2011 alone, and on average >3,000 unique visitors per day (based on IP addresses). This represents over 3 million hits per month. Google scholar (24 April 2012) found over 800 citations for “World Register of Marine Species” and that it was cited in over 100 publications.

**Table 8:** Some of the organisations using the WoRMS web service for their data management systems and/or research.

Country	Organisation
	Wikipedia, the free encyclopedia
Canada	University of British Columbia
Denmark	European Environment Agency, EUNIS
Denmark	International Council for the Exploration of the Sea (ICES)
EU	EMODNet Pilot Portal for Biology
Finland	HELCOM, Helsinki
France	Centre de Recherche Halieutique Méditerranéenne et Tropicale (Institut de Recherche pour le Développement, IRD)
France	Agence des Aires Marines Protégées (Ministre de lcologie, du Dveloppement durable, des Transports et du Logement)
France	Station Biologique de Roscoff
France	Université de Bordeaux
Germany	MariLim Gesellschaft für Gewässeruntersuchung mbH
Italy	Circolo Attività Subacquee Chieri, Sezione Didattica, I
Italy	UN Food and Agriculture Organisation
Morocco	Institut National de Recherche Halieutique (INRH)
The Netherlands	ETI BioInformatics
The Netherlands	Natural History Museum Rotterdam
UK	British Oceanographic Data Centre (BODC)
UK	Ecospan Environmental Limited
UK	Gardline Marine Sciences Group
UK	Marine Life Information Network (MarLIN)
UK	University of Manchester
Ukraine	National Academy of Sciences of Ukraine, Institute of Biology of the Southern Seas
USA	Coral Reef Ecosystem Division of NOAA, Hawaii
USA	Encyclopedia of Life
USA	GenBank, NCBI
USA	International Institute for Species Exploration
USA	MIT Sea Grant College Program
USA	San Diego Supercomputer Center,
USA	United States Antarctic Program at the National Museum of Natural History

**Table 9:** Organisations with licensed copies of WoRMS for their research and/or data management.

**Australia:**	CReefs project, AIMS; Statistics, Australian Antarctic Division; Interim Register of Marine and Nonmarine Genera (IRMNG), CSIRO Marine and Atmospheric Research; Museum and Art Gallery of the Northern Territory.
**Belgium**:	MUMM.
**Canada:**	SeaLifeBase, Fisheries Centre University of British Columbia; BOLD, Marine Barcode of Life (MarBOL); FMAP, Dalhousie University.
**Denmark:**	Department of Marine Ecology, National Environmental Institute University of Aarhus; Electronic Catalogue of Names, GBIF; Global Names Index, GBIF; Dept. of Marine Ecology, National Environmental Research Institute, Aarhus University.
**France:**	IFREMER (AVANO; BIOCEAN; Centre de Nantes, Département EMH); Institut de recherche pour le développement (IRD); Inventaire national du Patrimoine naturel (INPN), MNHN; Serena Application, Réserves Naturelles de France; Ecole Navale, Brest; Station Biologique de Roscoff; Diveboard.com.
**Germany**:	SeSAM, Senckenberg Forschungsinstitut und Naturmuseum Frankfurt; Institute for Polar Ecology, University of Kiel; German Marine Monitoring Programme, Federal Environmental Agency; Bundesamt für Seeschifffahrt und Hydrographie (BSH).
**Greece**:	MedOBIS, HCMR.
**India**:	Central Marine Fisheries Research Institute; Indian OBIS node, Centre for Marine Living Resources & Ecology. Ministry of Earth Sciences.
**Ireland**:	Trinity College Dublin.
**Italy**:	University of Messina; Circolo attività Subacquee Chieri.
**Netherlands**:	BEAST Database, Institute for Marine Resources and Ecosystem Studies (IMARES); Animal Project, Wikipedia NL.
**New Zealand**:	Saatchi & Saatchi; Leigh Marine Laboratory, University of Auckland.
**Portugal:**	Bioactive compounds project, Centre for Marine and Environmental Studies (CESAM) & Department of Biology University of Aveiro.
**Saudi Arabia**:	Computational Bioscience Research Centre (CBRC), King Abdullah University of Science and Technology.
**South Africa**,	MyDiveAlbum.
**Spain:**	Fauna Ibérica Project, Universidade da Coruña; Instituto Mediterraneo de Estudios Avanzados (IMEDEA); Review on patented marine natural products, Juan J. González.
**Switzerland:**	GBIF Swiss Node; Musée cantonal de zoologie.
**Taiwan:**	Catalogue of Life in Taiwan (TaiBNET), Academia Sinica. Marine Ecological Solutions Ltd; School of Computer Science University of Manchester, and National Centre for Text Mining (NaCTeM) Manchester Interdisciplinary Biocentre; Plymouth Marine Laboratory; Divenation.com; Marine Scotland Science, Marine Laboratory; School of Ocean Sciences, Bangor University; Environment Department, University of York.
**UK:**	
**USA:**	ZipcodeZoo.com; SeamountsOnline, University of California San Diego; Ocean Genome Legacy; Porifera Tree of Life project (PorToL), University of Alabama at Birmingham; Multispecies fisheries models in the Gulf of Mexico and South Atlantic, Florida Fish and Wildlife Conservation Commission; NMFS-COPEPOD, National Marine Ecosystems Division, Oceanic and Atmospheric Administration (NOAA); Moorea Biocode Project, National Museum of Natural History Smithsonian Institution; US Environmental Protection Agency (US EPA), Coastal Ecology Branch; USGS Western Fisheries Research Center, Marine Hatfield Science Center.

### User feedback

Typical benefits of WoRMS to users were that: (1) the process of reconciling names was automated; (2) the entry of names in a database could use a drop-down menu of existing names from WoRMS, so errors in manual entry could be avoided; (3) the names followed a standardized taxonomic hierarchy, thus aiding a user's classification of species in their own database and publications; (4) it was a single standard authoritative and time-saving resource to reference names and their classification; (5) it has an efficiently responding editorial system (Neil Holdsworth, personal communication, 25 November 2010); and (6) checking of names from collaborators and the literature could be automated. Including researching names not in WoRMS that would need to be checked from other sources, WoRMS saved users significant time compared to manually checking names using search engines and the literature (e.g. 14 times less time, Karen Stocks personal communication, 18 November 2010). Thus the availability of WoRMS not only saves users time but will improve quality control in the use of marine species names. WoRMS is also used as a naming standard for semantic frameworks used in databases for different projects (Roy Lowry, personal communication, 4 November 2010).

## Discussion

WoRMS was formally launched to the world media in 2008. A press release in collaboration with the Census of Marine Life resulted in remarkable media uptake in 27 countries and nine languages. By June 2008, it was covered in 298 online stories, 23 newswires, 23 newspapers, and interviews on eight radio and two television stations. This was impressive for an online biodiversity database, and reflected the great media and popular interest in discoveries of marine biodiversity found by the Census of Marine Life [87].

The development of WoRMS has accelerated the availability of taxonomically authoritative GSDs for Species 2000, OBIS and GBIF. A growing number of GSDs are provided to Species 2000, sometimes replacing earlier GSDs that are no longer updated. In addition to WoRMS being directly provided to other organisations, many more use WoRMS GSDs via Species 2000 and its Catalogue of Life (CoL), and through the EOL website. Uniquely amongst species name systems, WoRMS provides environmental context (i.e. marine) for species. The European component of WoRMS, ERMS, has been recommended as a standard when using species names in the European Union under the Infrastructure for Spatial Information in the European Community (INSPIRE) Directive.

### Benefits

Some of the most important benefits of WoRMS will be improved taxonomic efficiency, and greater quality control in the use of species names in the wider literature and environmental management. For example, EurOBIS corrected 28% of the names in its database by using WoRMS [88]. By making a single inventory of all marine species names easily accessible on the internet it is anticipated that people will use it to correct spelling mistakes, use the currently accepted names rather than synonyms, and bring omissions, errors and anomalies to the attention of the taxonomic editors to address. The authors of popular species identification guides will find it easy to update the species names they use, and ecologists, conservationists and environmental managers will be using species names more consistently. The increasing usage of WoRMS indicates this is happening.

Taxonomic research will also benefit. Duplicate descriptions of the same species will be reduced because researchers will have a checklist of related species to compare their specimens and observations with, and contact details of experts to discuss their findings with. Authors of species descriptions can check if similar names are already in use, and thus may choose more unique names and avoid homonyms. The production of WoRMS has added benefits in fostering collaboration between experts at a global scale. Easy access to the register allows ecologists and local observers to correct their use of taxonomic names. In turn, this stimulates biogeographic and evolutionary research.

### Use in research

Although initially established to provide open-access information on marine species nomenclature, the aggregation of so much content is providing unanticipated benefits to researchers. ERMS provided the basis for (1) a review of taxonomic expertise and resources, including a list of species identification guides [89], [90], and (2) an analysis of trends in species discoveries and predictions of how many more species remain to be discovered [17], [91]. This research stimulated the development of a new statistical approach, unusual in that it allowed calculation of confidence limits, to predict species richness from past rates of discovery [92]. This work was then extended to WoRMS and CoL to predict global species richness [22]. Other approaches to estimate species richness used the rate of discovery of higher taxa in WoRMS and other databases [21], and developed a software tool to provide a structured approach to using expert knowledge to estimate richness [93]. WoRMS has also contributed to the annual reports of species discoveries [94], [95]. Fisher et al. [96] matched 2,380 species names from WoRMS to a bibliographic database so as to identify bias in research on coral, kelp, seagrass and mangrove habitats.

Groups of WoRMS taxonomic editors have begun to synthesise knowledge on their taxon, including a major collaborative paper co-authored by over 100 editors [91]. These studies form the basis for a special collection of papers in PLoS ONE. To date, they review the global diversity of several taxa: (1) Crustacea: Remipedia [97], Monstrilloida copepods [98], Tanadiacea [99], and non-asellote isopods [100], [101]; (2) Cnidaria: Stylasteridae corals [102] and Pennatulacea corals [103]; (3) Echinodermata: Ophiuroidea [104] and Asteroidea [105]; as well as (4) Porifera [106] Ascidacea [107]; Oligotrichea protists [108]; Reptilia [109]; and Placozoa [110]. The present paper provides the introduction and context for this collection. It complements other PLoS ONE collections, notably those from the Census of Marine Life, e.g., [111] and one paper fits two collections [112]. The study synthesises how many species are described, the number of accepted species names and synonyms, estimates of how many molecular cryptic species may exist, how many undescribed species are already in specimen collections, how many undescribed species have been found in field samples, and predictions of how many more species may yet be discovered [91]. This study provides a baseline of current knowledge of marine biodiversity at the species level, summarises the rate of progress in discovering species, and should be reviewed every few years.

### Future prospects

Several initiatives are underway within WoRMS but not yet visible. These include new GSDs websites, and Thematic Species Databases on introduced species and parasite-host relationships. The major taxonomic gaps are amongst Mollusca, but no doubt there are omissions in other taxa and continuing updates needed. In the absence of alternative infrastructure and for taxonomic convenience, editors may add freshwater and terrestrial relatives to their marine GSDs, as already the case for Copepoda, Isopoda, Porifera, Gastrotricha, and Tardigrada. Users are encouraged to contact editors regarding possible omissions and errors in the database content. Continual improvements to content and database functionality are required. For example, about 5% of the literature sources are estimated to be duplicate entries and need to be manually rationalised. Species' fossil status is being categorised using a standard stratigraphy following a proposal from the editors for Foraminifera and Echinoidea. Linking of literature references to electronic copies of the publication is being implemented through hosting documents within WoRMS, and linking out to sources, such as the Biodiversity Heritage Library. Thus the content continually expands at the initiative of editors, or users, and may be funded by special projects with particular research goals.

Users may like all content on one page but this is increasingly being provided from different experts (e.g. taxonomist, ecologist, biogeographer). Thus developments can present challenges for web page design, distinguishing which experts have validated which content, agreement on controlled vocabularies, and patience to reconcile different perspectives. We expect greater linkage with species distribution data in OBIS and GBIF. Several editors have developed online species identification resources. The future may see an online guide to all marine species. Some species' conservation status is indicated and WoRMS updates species names for the IUCN Red List. Thus, there is potential to create a thematic database on marine species of special conservation interest. New tools and online resources are materialising that provide opportunities for WoRMS to be more interoperable with online journals (e.g. using DOI or other identifiers), and other databases; such as the FilterPush (http://etaxonomy.org/mw/FilteredPush) that networks species names.

The classification of species by their biological (e.g. body size, parasites, dispersal), ecological (e.g. habitat), and other (e.g. invasive, threatened) attributes, has a multiplier effect on the potential research and user audience for WoRMS. Already there are improvements in the ability to sample and analyse marine species. As a consequence of WoRMS, we are already witnessing improved communication within the scientific community, and anticipate increased taxonomic efficiency and quality control in marine biodiversity research and management.
